# Advances in Molecular Mechanisms and Precision Interventions of Cardiovascular Injuries Related to Glucose and Lipid Metabolism Disorders

**DOI:** 10.31083/RCM42406

**Published:** 2025-10-17

**Authors:** Li Wu, Jiyao Xu, Chenxia Wu, Minyan Wang, Tingting Chen, Conghua Ji, Wei Mao

**Affiliations:** ^1^The First School of Clinical Medicine, Zhejiang Chinese Medical University, 310053 Hangzhou, Zhejiang, China; ^2^Department of Cardiology, Shanxi Cardiovascular Disease Hospital, 030027 Taiyuan, Shanxi, China; ^3^Cardiovascular Department, Zhejiang Hospital (Affiliated Zhejiang Hospital, Zhejiang University School of Medicine), 310030 Hangzhou, Zhejiang, China; ^4^School of Public Health, Zhejiang Chinese Medical University, 310053 Hangzhou, Zhejiang, China; ^5^Zhejiang Key Laboratory of Integrative Chinese and Western Medicine for Diagnosis and Treatment of Circulatory Diseases, Zhejiang Hospital, 310030 Hangzhou, Zhejiang, China

**Keywords:** glucose and lipid metabolism disorders, cardiovascular disease, atherosclerosis, molecular mechanisms, precision medicine, metabolic memory, *cGAS*-*STING*

## Abstract

This article reviews the latest research progress (2018–2025) on the molecular mechanisms linking glucose and lipid metabolism disorders (GLMDs) to cardiovascular injury, specifically atherosclerotic cardiovascular disease (ASCVD), diabetic cardiomyopathy (DbCM), heart failure (HF), and cardiac autonomic neuropathy (CAN). This review employed a targeted analysis of key publications from the PubMed, Web of Science, and EMBASE databases, as well as citation tracking, prioritizing molecular pathways and interventions for these four complications. The key mechanisms include: metabolic inflammation: the advanced glycation end products (AGEs)–receptor of AGE (RAGE) axis activates NF-κB, promotes vascular cell adhesion molecule-1 (VCAM-1)/monocyte chemoattractant protein-1 (MCP-1) overexpression, and accelerates monocyte infiltration; myocardial lipotoxicity: CD36 mediates fatty acid overload → mitochondrial damage → cyclic guanosine monophosphate-adenylate synthetase (*cGAS*)-STING pathway activation → myocardial apoptosis; metabolic memory: hyperglycemia continuously releases small extracellular vesicle (sEV) miR-15-16 clusters through the O-GlcNAc–CaMKIIδ–STAT1 loop, mediating remote myocardial injury; gut–heart axis disorder: Trimethylamine N-Oxide (TMAO) promotes thrombosis and endothelial injury. Precision strategies based on the above mechanisms, such as SGLT2 inhibitors to improve myocardial energy metabolism, targeting acyl-coenzyme A binding protein (ACBP)/TGR5 to alleviate lipotoxicity, and microbiota regulation, have demonstrated potential in clinical research. Future focus should include (1) GLMD heterogeneity typing; (2) tissue-targeted delivery system; (3) multi-omics–AI dynamic risk modeling.

## 1. Introduction

The global prevalence of diseases related to glucose and lipid metabolism disorders (GLMDs) (such as diabetes, metabolic fatty liver disease, and obesity) continues to rise [1], and cardiovascular complications caused by GLMDs have become the leading cause of death [2, 3]. The core pathological features of GLMDs include insulin resistance (IR) and ectopic lipid deposition [4], and their occurrence is the result of the combined effects of genetic susceptibility, environmental factors (such as diet and lack of exercise), and epigenetic regulation [5]. The key pathological processes involve insulin signal transduction disorders (such as IRS/PI3K/Akt pathway inhibition) [6], neuroendocrine regulation imbalance (such as hypothalamic-pituitary-adrenal axis (HPA axis) activation, increased sympathetic nerve tone) [3], redox homeostasis disruption (excessive production of reactive oxygen species (ROS)) [7], persistent low-grade inflammatory response [8], and intestinal microecological disturbances [2, 9, 10, 11]. These processes interact with each other to form a vicious cycle, ultimately leading to vascular endothelial damage, myocardial remodeling and abnormal neural regulation, significantly increasing the risk of atherosclerotic cardiovascular disease (ASCVD), heart failure (HF) and cardiac autonomic neuropathy (CAN) [12]. Studies have shown that long-term exposure to high blood sugar and high triglyceride environments significantly increases the risk of cardiovascular events such as myocardial infarction and stroke [13, 14]. An elevated triglyceride-glucose index (TyG index) is significantly associated with an increased risk of cardiovascular events [15, 16]. It is worth noting that although traditional risk factor management (such as glucose lowering and lipid regulation) can partially reduce the risk of cardiovascular disease (CVD), a large number of patients (especially those with good blood sugar control) still have high residual risks [17, 18], highlighting the urgency of in-depth exploration of the deep molecular mechanisms of GLMD-induced cardiovascular damage and the development of precise intervention strategies. The advancement of multi-omics technologies (genomics, transcriptomics, proteomics, metabolomics, microbiome) provides powerful tools for systematic analysis of these mechanisms [9, 19].

Traditional risk management has a limited effect on the residual cardiovascular risk of GLMD patients, highlighting the urgency of exploring deep molecular mechanisms and developing precision interventions. This article aims to review the progress of the molecular mechanisms of GLMD-related cardiovascular damage (ASCVD/diabetic cardiomyopathy (DbCM)/CAN), with a special focus on emerging pathways such as metabolic memory, lipotoxicity and microbiota-host dialogue, and evaluate the clinical translation potential of targeted intervention strategies.

## 2. Mechanisms of Cardiovascular Injury in GLMD: Molecular Network Analysis

### 2.1 Metabolic Inflammation and Endothelial Dysfunction: From AGEs to Epicardial Adipose Tissue (EAT)

Chronic low-grade inflammation (metabolic inflammation, Metaflammation) is the core link between GLMD and ASCVD [20]. Hyperglycemia, elevated free fatty acids (FFA) and advanced glycation end products (AGEs) jointly activate the inflammatory pathway of endothelial cells (ECs), destroying the barrier function and promoting monocyte adhesion and infiltration and foam cell formation.

#### 2.1.1 The Core Role of the AGEs-RAGE-NF-κB Axis

Sustained hyperglycemia leads to the massive generation of AGEs. AGEs bind to the ECs surface receptor RAGE, triggering a strong activation of the downstream NF-κB signaling pathway [21], which leads to the overexpression of proinflammatory factors such as vascular cell adhesion molecule-1 (VCAM-1) and monocyte chemoattractant protein-1 (MCP-1). The upregulation of VCAM-1/MCP-1 promotes the migration of monocytes to the subendothelium of blood vessels and their differentiation into macrophages, which ingest oxidized low-density lipoprotein (oxLDL) and transform into foam cells, forming the lipid core of atherosclerotic plaques [22]. Hyperglycemia activates the NF-κB pathway and also promotes the release of proinflammatory factors such as tumor necrosis factor-α (TNF-α) and interleukin-6 (IL-6). At the same time, hyperglycemia inhibits the phosphorylation of endothelial nitric oxide synthase (eNOS) Ser1177, reduces the production of nitric oxide (NO), and leads to vasodilation disorders [2]. Clinical studies have shown that serum IL-6 levels are significantly elevated in patients with type 2 diabetes mellitus (T2DM) [23].

#### 2.1.2 Multiple Strikes of Lipoprotein Abnormality

Lipoprotein(a) [Lp(a)]: Lp(a) has the “dual identity” of promoting atherosclerosis and thrombosis. Its apolipoprotein(a) [apo(a)] structure is highly homologous to plasminogen and can competitively inhibit plasminogen activation, weakening fibrinolytic activity [24, 25]. A Multinational prospective cohort study showed that high Lp(a) level (≥90th percentile) increased the risk of ASCVD by 46% (hazard ratio (HR) = 1.46), and the risk of diabetes subgroup further increased to 92% (HR = 1.92) [26].

#### 2.1.3 Abnormal Function of High-Density Lipoprotein (HDL)

In the GLMD state, HDL undergoes oxidation, inflammation and other modifications, such as nitration of apolipoprotein A-I and accumulation of serum amyloid A, leading to impaired cholesterol reverse transport (RCT) ability (ATP binding cassette transporter A1/G1 pathway disorder); The anti-inflammatory and antioxidant functions significantly decreased (inactivation of paraoxonase 1 and increase in pro oxidative LDL); Loss of endothelial protective function (reduced eNOS activation and insufficient NO production) [27]. Studies have shown that the cholesterol reverse transport capacity of HDL in diabetes patients is controversial, but its antioxidant function is clearly impaired [28, 29].

#### 2.1.4 The Role of EAT as an “Active Endocrine Organ” 

EAT is a fat pad that is close to the myocardium and coronary arteries. It undergoes significant pathological changes under the GLMD state, manifested by adipocyte hypertrophy, macrophage infiltration (increase in proinflammatory M1 type) [30], increased secretion of proinflammatory factors (such as TNF-α, IL-6, chitinase 3-like protein 1 YKL-40) and decreased secretion of anti-inflammatory factors (such as adiponectin, C1q/tumor necrosis factor-related protein 9 CTRP9, Metrnl) [31]. The inflammatory factors and FFA secreted by EAT can directly infiltrate the underlying myocardial tissue and coronary artery wall, and promote local inflammation, oxidative stress, vascular smooth muscle cell proliferation and plaque instability through paracrine effects [31]. In addition, EAT Hypoxia induces activation of hypoxia-inducible factor-1α (HIF-1α), abnormally upregulating vascular endothelial growth factor (VEGF) expression, promoting increased vascular permeability and myocardial microvascular leakage, and aggravating myocardial edema and fibrosis [32]. EAT thickness is positively correlated with the degree of coronary atherosclerosis [31]. Saturated fatty acids (such as palmitic acid) and lipopolysaccharide (LPS) synergistically activate macrophage TNF-α secretion, inhibit adipocyte metabolic function, and aggravate insulin resistance and vascular endothelial inflammation [33].

**Fig. 1.**
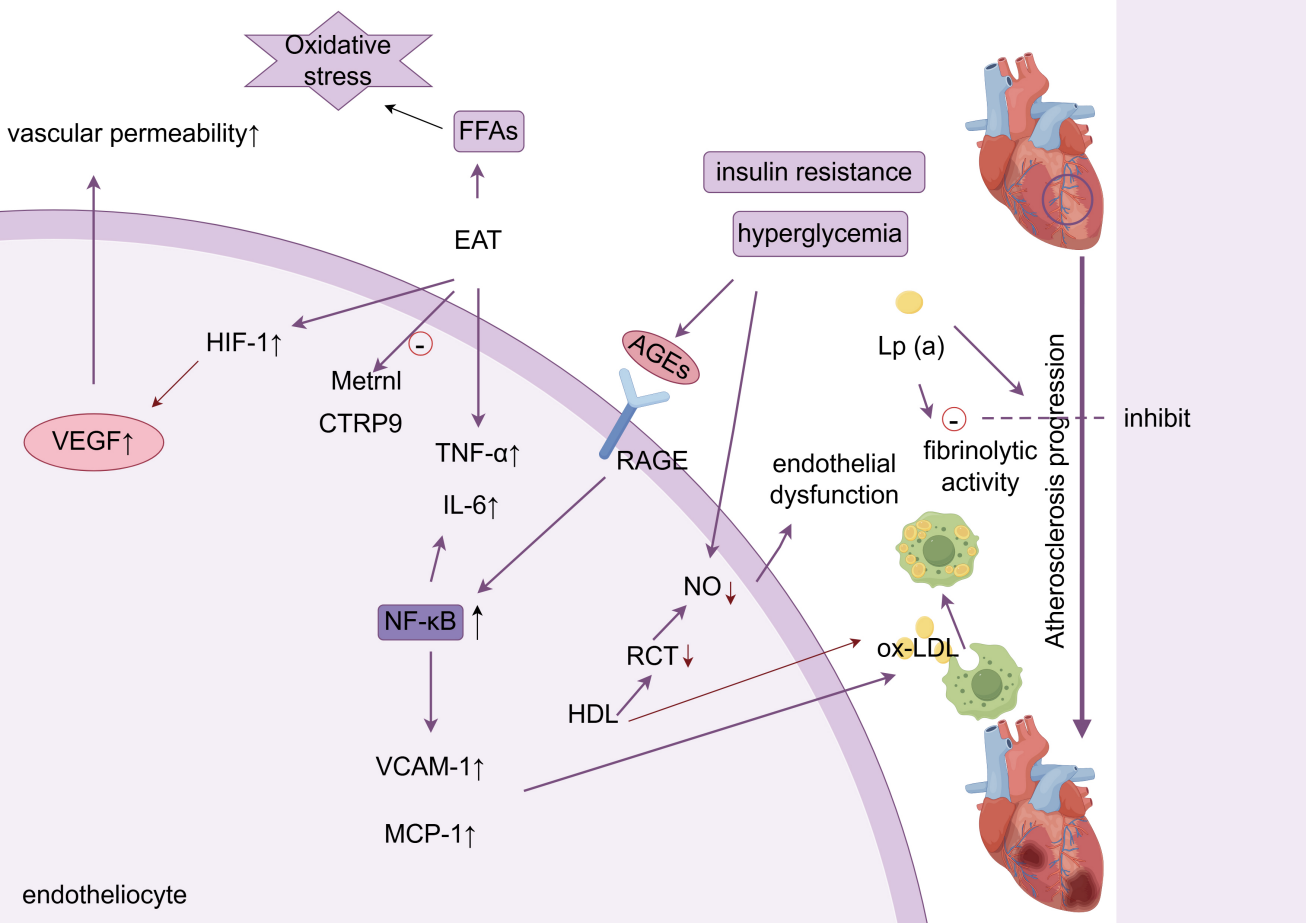
**Metabolic inflammation and endothelial dysfunction**. ox-LDL, oxidized low-density lipoprotein; AGEs, advanced glycation end products; VEGF, vascular endothelial growth factor; RCT, cholesterol reverse transport; RAGE, receptor for advanced glycation endproducts; NO, nitric oxide; FFA, free fatty acids; HIF-1, hypoxia-inducible factor-1; EAT, epicardial adipose tissue; TNF-α, tumor necrosis factor-α; IL-6, interleukin-6; Lp(a), Lipoprotein(a); HDL, high-density lipoprotein; VCAM-1, Vascular Cell Adhesion Molecule-1; MCP-1, Monocyte Chemoattractant Protein-1. Arrows indicate the direction of change in the glucolipid metabolic disorder (GLMD) state: up arrows (↑) signify promotion, increase, or upregulation; down arrows (↓) represent inhibition, decrease, or downregulation. The figure was created with figdraw (https://www.figdraw.com/).

These synergistic interactions are schematically summarized in Fig. 1.

### 2.2 Myocardial Energy Metabolic Remodeling and Lipotoxicity: From CD36 to cGAS STING

GLMD (especially diabetes) leads to a pathological change in the myocardial energy metabolism pattern, which is characterized by excessive reliance on fatty acid oxidation (FAO) for energy supply, impaired glucose utilization, mitochondrial dysfunction and accumulation of toxic lipid intermediates, collectively referred to as “lipotoxicity”, which is the core mechanism of DbCM [34, 35].

#### 2.2.1 Metabolic Substrate Conversion Imbalance and CD36 Regulation

In physiological conditions, the heart has metabolic flexibility and can switch between glucose and fatty acid oxidation according to substrate supply. In GLMD, impaired myocardial insulin signaling leads to reduced translocation of glucose transporter 4 (GLUT4) to the cell membrane, and myocardial glucose uptake and utilization are significantly reduced [36, 37]. As compensation, myocardial cells increase fatty acid uptake and oxidation. Fatty acid transporter *CD36* plays a key role in this process. Studies have revealed the regulation of *CD36* by the bile acid-*TGR5* pathway: In the diabetic state, the level of bile acids (especially deoxycholic acid DCA) that activate *TGR5* in plasma is reduced. *TGR5* is a G protein-coupled bile acid receptor expressed in cardiomyocytes. Absence of *TGR5* signaling (e.g., cardiomyocyte-specific *Tgr5* knockout mice) or attenuation (diabetic state) promotes palmitoylation of *CD36* protein by upregulating the expression and activity of palmitoyltransferase *DHHC4*. Enhanced palmitoylation of *CD36* increases its translocation to the cell membrane, significantly increasing fatty acid uptake by cardiomyocytes. This *CD36* Uncontrolled membrane translocation is an important mechanism of myocardial lipid overload [34].

#### 2.2.2 Mitochondrial Dysfunction and ROS Burst

The influx of excess fatty acids exceeds the mitochondrial β-oxidation capacity, leading to the accumulation of incomplete oxidation intermediates such as acylcarnitine [38]. At the same time, FAO enhancement is accompanied by increased production of reactive oxygen species (ROS) in the electron transport chain [39]. In metabolic disorders such as IR or diabetes, the antioxidant defense system (such as superoxide dismutase SOD and glutathione system) is often damaged, leading to increased oxidative stress [40]. Increased levels of lipid peroxidation products (such as malondialdehyde (MDA)) are a sign of myocardial oxidative damage [41].

#### 2.2.3 Dysregulation of the Autophagy-Lysosome System

Autophagy is a key process for clearing damaged organelles (such as dysfunctional mitochondria) and misfolded proteins. In diabetic myocardium, autophagic flux is often impaired, manifested by decreased expression of key autophagic proteins (such as Beclin-1, microtubule-associated protein 1 light chain 3-II (LC3-II)) or impaired fusion of autophagosomes with lysosomes [42].

#### 2.2.4 Mitochondrial DNA Leakage and cGAS-STING Pathway Activation

Severe or persistent mitochondrial damage can lead to the release of mitochondrial DNA (mtDNA) into the cytoplasm. The mtDNA in the cytoplasm is recognized by the DNA sensor cyclic guanosine monophosphate-adenylate synthetase (cGAS), which activates the adaptor protein stimulator of interferon genes (*STING*). Activated *STING* then recruits and activates TANK binding kinase 1 (TBK1), phosphorylates the transcription factor interferon regulatory factor 3 (IRF3), and ultimately induces the production of proinflammatory cytokines such as type I interferon (such as IFN-β). This pathway has been confirmed to be an important bridge connecting mitochondrial damage, inflammatory response and myocardial fibrosis in DbCM. Animal experiments have shown that specific knockout of the *Sting* gene in cardiomyocytes can significantly inhibit this pathway, reduce IFN-β release, and improve cardiac function [43, 44].

High glucose and lipotoxic environment enhance *cGAS*-*STING* activation: Enhanced mitochondrial damage: A hyperglycemic environment increases ROS production, leading to mitochondrial dysfunction and oxidative damage to mtDNA, and increasing mtDNA leakage [45]. Lipotoxicity further exacerbates mitochondrial damage. The *cGAS-STING* pathway drives the myocardial inflammatory cascade through the TBK1-IRF3-IFNβ axis, forming a closed loop of “lipotoxicity → mitochondrial damage → *cGAS*-*STING* → inflammation”. This closed loop is one of the core characteristics that distinguish DbCM from non-diabetic myocardial injury.

acyl-coenzyme A binding protein (ACBP) and myocardial lipotoxicity: In diabetes cardiomyopathy, ACBP aggravates myocardial lipotoxicity by regulating fatty acid metabolism and affecting myosin function. ACBP disrupts the contractile structure by binding to the sarcomere protein MyBPC3. The upregulation of ACBP leads to disturbances in fatty acid metabolism, further triggering oxidative stress and mitochondrial damage, ultimately resulting in myocardial energy metabolism remodeling and apoptosis. Myocardial specific knockout of ACBP can improve cardiac function in diabetes mice, suggesting that ACBP is a potential double effect target [46].

Lipid droplet dynamic imbalance and ceramide toxicity: When the fatty acid uptake rate far exceeds the oxidation capacity, neutral triglycerides (TG) are stored in myocardial cells in the form of lipid droplets. Moderate lipid droplet formation has a protective effect in buffering lipotoxicity, but this effect is limited. In severe GLMD, myocardial lipid droplets are excessively deposited and lipid droplet mobilization and utilization are blocked. In addition, saturated fatty acids (such as palmitic acid) can be converted into ceramide, a highly cytotoxic sphingolipid. Ceramide promotes macrophage inflammatory response and plaque instability by activating specific receptors (such as cysteine leukotriene receptor 2 CYSLTR2, purinergic receptor P2RY6), exacerbating atherosclerosis [47]. In addition, ceramide further exacerbates metabolic disorders by inhibiting the insulin signaling pathway and mitochondrial metabolism. AMP-activated protein kinase (AMPK) is a key kinase that regulates energy metabolism. Its activation (such as by drugs or exercise) can promote FAO and significantly reduce ceramide accumulation, and is an important target for improving lipotoxicity [48].

In summary, lipid toxicity is mediated by *CD36* mediated fatty acid overload, mitochondrial ROS burst, and activation of the c*GAS STING* pathway, forming a vicious cycle of ‘lipid oxidative stress inflammation’, which is the core driving mechanism of DbCM. Recent studies have further revealed the interaction between mitochondrial quality control imbalance and lipid toxicity, suggesting that the mitochondrial deacetylase SIRT4 may participate in lipid toxicity processes by regulating *CD36* palmitoylation and mitochondrial autophagy, providing a new perspective for targeting mitochondrial lipid metabolism interactions.

**Fig. 2.**
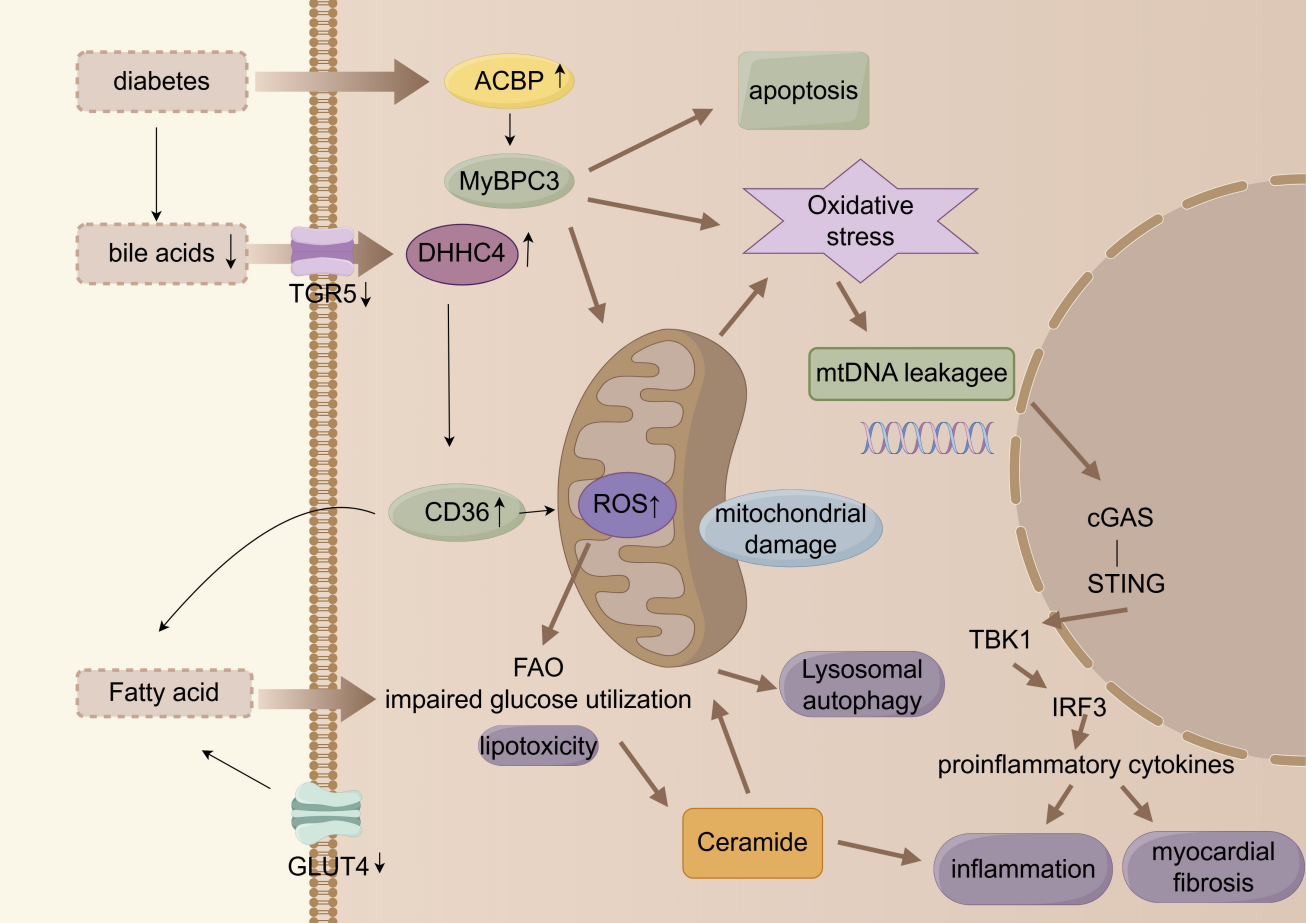
**Myocardial energy metabolic remodeling**. cGAS, cyclic guanosine monophosphate-adenylate synthetase; FAO, fatty acid oxidation; ACBP, acyl coenzyme A binding protein. Arrows indicate the direction of change in the glucolipid metabolic disorder (GLMD) state: up arrows (↑) signify promotion, increase, or upregulation; down arrows (↓) represent inhibition, decrease, or downregulation. The figure was created with figdraw (https://www.figdraw.com/).

The multi-level metabolic alterations are illustrated in Fig. 2.

### 2.3 Hyperglycemia “Metabolic Memory” and Remote Damage Mediated by Extracellular Vesicles

Clinical observations (such as the Diabetes Control and Complications Trial/Epidemiology of Diabetes Intervention and Complications Study (DCCT/EDIC)) have confirmed that even if blood sugar is well controlled in the later stage, previous exposure to high blood sugar can still lead to the continued progression of cardiovascular complications of diabetes. This phenomenon is called “metabolic memory” or “hyperglycemia memory” (Hyperglycemic Memory, HGM) [49, 50]. Its molecular mechanism involves multi pathway synergistic effects: O-GlcNAc glycosylation/CaMK2a positive feedback loop: Hyperglycemia increases protein O-linked N-acetylglucosamine via the hexosamine biosynthesis pathway (HBP) (O-GlcNAc) modification. O-GlcNAc modification specifically activates the calcium/calmodulin-dependent protein kinase IIδ (CaMKIIδ) of endothelial cells, forming a self-sustaining loop: O-GlcNAc modification directly acts on the Thr306 site of CaMKIIδ to enhance its stability. Activated CaMKIIδ inhibits O-GlcNAc hydrolase (OGA) expression, further increasing overall O-GlcNAc levels [51].

Sustained release of the sEV *miR-15-16* cluster: Activated CaMKIIδ phosphorylates and activates the transcription factor signal transducer and activator of transcription 1 (STAT1). STAT1 translocates into the nucleus, binds to the promoter region of the miR-15a/16-1 gene cluster, and continuously promotes its transcription. These miRNAs are packaged into extracellular vesicles (sEVs) secreted by endothelial cells and released [51, 52].

Forkhead box protein O1 (FoxO1)-mediated cardiomyocyte apoptosis: Endothelial-derived sEVs enriched in the *miR-15-16* cluster are taken up by cardiomyocytes, and *miR-15-16* targets, and suppresses Serum/Glucocorticoid Regulated Kinase 1 (SGK1), Proto-OncogeneSerine/Threonine Kinase (RAF1), and Vascular Endothelial Growth Factor A (VEGFA), reducing Akt phosphorylation (p-Akt) and thereby decreasing inhibition on the transcription factor FoxO1. This results in significantly increased FoxO1 expression, ultimately inducing cardiomyocyte apoptosis and cardiac dysfunction [51].

**Fig. 3.**
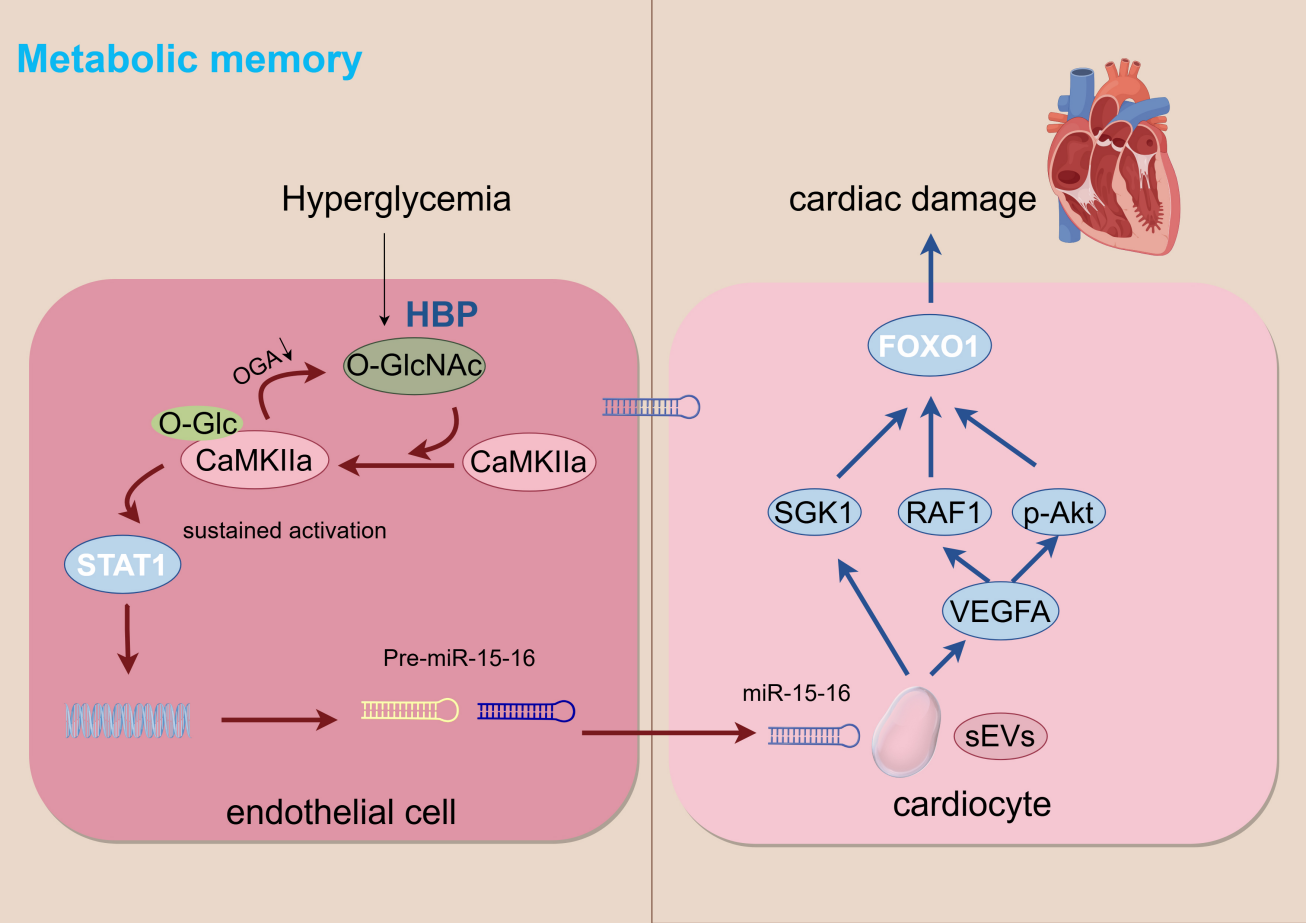
**Metabolic memory**. HBP, hexosamine biosynthesis pathway. Arrows indicate the direction of change in the glucolipid metabolic disorder (GLMD) state: down arrows (↓) represent inhibition, decrease, or downregulation. The figure was created with figdraw (https://www.figdraw.com/).

Clinical verification and significance: In the plasma sEV of diabetes patients with heart failure, the level of *miR-15-16* was significantly increased, and was significantly negative with left ventricular ejection fraction (LVEF). This indicates that sEV *miR-15-16* is an independent biomarker of cardiac dysfunction independent of current blood glucose status and a key mediator of metabolic memory induced sustained myocardial injury. This study emphasizes the importance of early reinforcement of glucose control or inhibition of O-GlcNAc modification in blocking metabolic memory circuits. The metabolic memory of hyperglycemia is illustrated in Fig. 3.

### 2.4 CAN

CAN is a serious complication of GLMD, characterized by sympathetic/parasympathetic nerve imbalance, manifested by decreased heart rate variability (HRV), resting tachycardia, postural hypotension and painless myocardial ischemia, significantly increasing the risk of malignant arrhythmias and sudden cardiac death. Its pathogenesis is closely related to multiple factors such as chronic hyperglycemia-induced oxidative stress, visceral fat inflammation and autoimmune attack. Early identification of CAN requires the combination of functional imaging and dynamic biomarker detection [53, 54].

#### 2.4.1 Assessment of Autonomic Function and Sympathetic Dominance

HRV analysis through a 24-hour dynamic electrocardiogram is the gold standard for diagnosing CAN. The low frequency/high frequency power ratio (LF/HF) of diabetic patients is significantly higher than that of healthy people, which clearly indicates that the sympathetic nerve tone is dominant over the parasympathetic nerve [53, 55].

#### 2.4.2 Visceral Fat Inflammation Drives Neural Damage

Visceral adipose tissue (VAT) is an important source of inflammation and FFA. Under GLMD, VAT releases a large amount of FFA and proinflammatory factors (such as TNF-α, IL-1β). FFA can induce downstream inflammatory signaling (such as NF-κB) activation by activating Toll-like receptor 4 (TLR4) located on neurons and glial cells [56, 57].

#### 2.4.3 Potential Role of Autoimmune Factors

The prevalence of CAN is significantly higher in patients with autoimmune diabetes (such as latent autoimmune diabetes in adults LADA and classic type 1 diabetes). A cross-sectional study showed that the prevalence of CAN in patients with positive anti-islet cell antibodies (ICA) was significantly higher than that in the ICA-negative group [57].

### 2.5 Gut Microbiota Dysbiosis and Its Metabolites

Gut microbiota and its metabolites constitute a complex “gut-heart axis” that plays an increasingly clear role in GLMD-related cardiovascular damage [11]. These metabolites affect cardiovascular health through multiple mechanisms, including regulation of inflammatory responses, lipid metabolism, and insulin sensitivity.

Harmful Metabolites:

Trimethylamine oxide (TMAO): Gut microbiota metabolizes choline and carnitine in food to produce trimethylamine (TMA), which is oxidized to TMAO by the liver enzyme flavin monooxygenase 3 (FMO3). High TMAO levels are independently associated with increased risk of ASCVD and HF. TMAO promotes atherosclerosis by promoting foam cell formation, enhancing platelet reactivity (increasing thrombotic risk), and inducing endothelial dysfunction [58, 59].

#### 2.5.1 Secondary Bile Acid Dysregulation

GLMD is often accompanied by changes in intestinal flora, which affects bile acid metabolism. The abnormal increase in the proportion of visceral fat (such as deoxycholic acid DCA) may promote inflammation or affect farnesoid X receptor (FXR)/*TGR5* signaling is involved in cardiovascular damage [34, 60].

#### 2.5.2 Reduction of Beneficial Metabolites

Short chain fatty acids (SCFAs): SCFAs (such as acetate, propionate, and butyrate) are produced by fermentation of dietary fiber through gut microbiota. They have anti-inflammatory properties, maintaining intestinal barrier integrity, and improving insulin sensitivity [61]. GLMD status is often accompanied by a decrease in the abundance of SCFA producing bacteria (such as Faecalibacterium prausnitzii and Roseburia), a decrease in SCFA levels, and a weakening of their cardiovascular protective effects [62, 63].

#### 2.5.3 Dysbiosis and Endotoxin Entry Into the Blood

Intestinal flora disorder (dysbiosis) is often accompanied by impaired intestinal barrier function (“leaky gut”), which leads to the translocation of bacterial endotoxin LPS into the blood circulation. LPS and FFA synergistically promote macrophage M1 polarization and drive TNF-α-dependent inflammatory cascade [32]. LPS is a potent TLR4 agonist that can trigger systemic low-grade inflammation and insulin resistance, indirectly promoting cardiovascular disease.

**Fig. 4.**
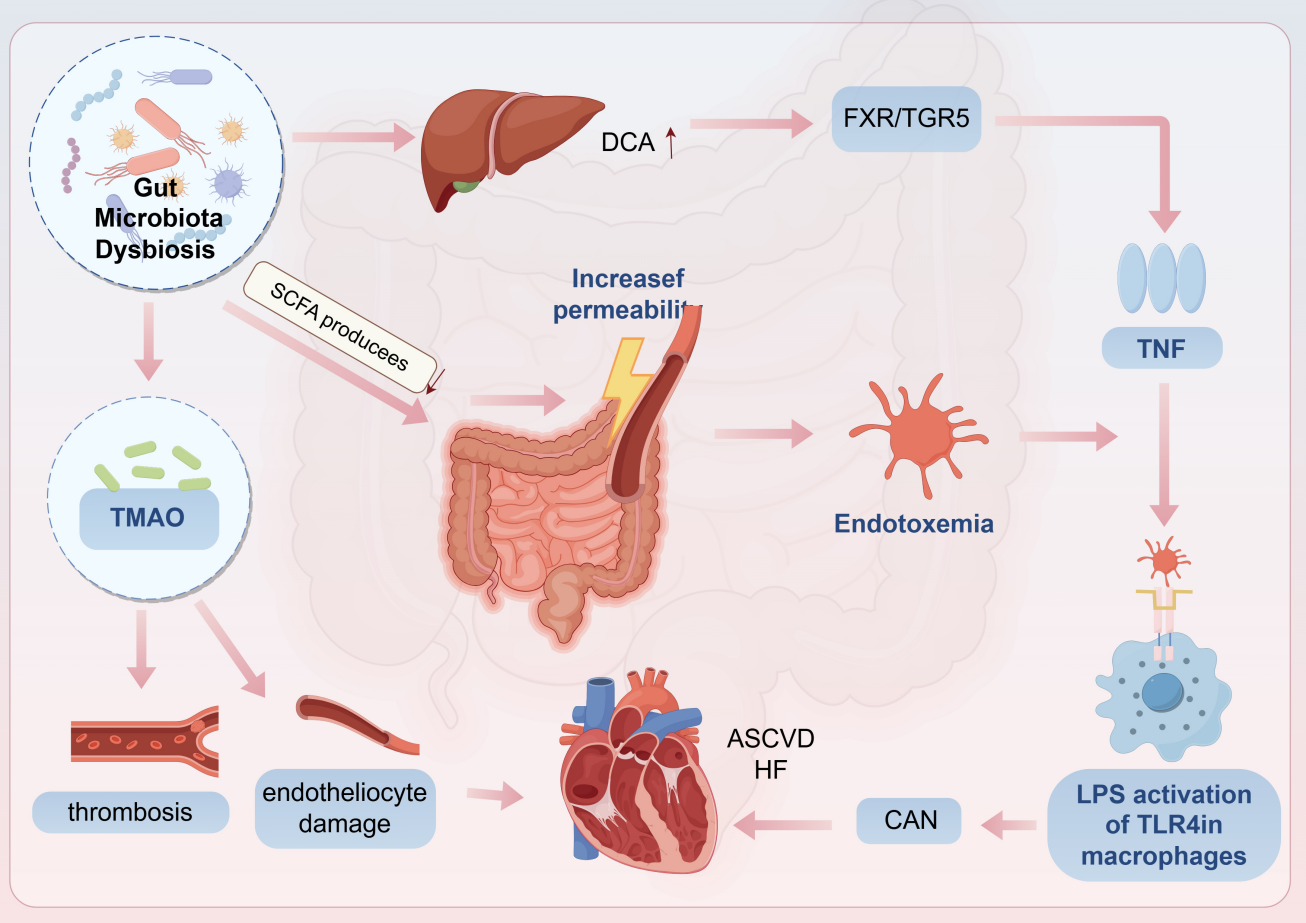
**CAN and Gut microbiota dysbiosis**. CAN, cardiac autonomic neuropathy; DCA, deoxycholic acid; TNF, tumor necrosis factor; HF, heart failure; ASCVD, atherosclerotic cardiovascular disease. Arrows indicate the direction of change in the glucolipid metabolic disorder (GLMD) state: up arrows (↑) signify promotion, increase, or upregulation. The figure was created with figdraw (https://www.figdraw.com/).

The gut-brain-heart axis dysregulation is visualized in Fig. 4.

## 3. Epidemiological Associations Between GLMD and Cardiovascular Events

### 3.1 Risk Amplification Effect

The association between GLMD and cardiovascular events exhibits a significant dose-response relationship and risk amplification. The risk of cardiovascular events in patients with diabetes is 2–4 times (male) to 2–7 times (female) higher than that in non-diabetic patients, and the absolute risk increases significantly with the younger age of diabetes (onset <40 years old) and the longer duration of the disease [64]. The variation of glycated hemoglobin (HbA1c) and the risk of major adverse cardiovascular events (MACE): Elevated HbA1c levels are an independent predictor of cardiovascular events: for every 1% increase in baseline HbA1c (b-HbA1c), the risk of severe coronary artery stenosis increases by 15% (odds ratio (OR) = 1.15, *p* = 0.046) [65]. When HbA1c (f-HbA1c) is ≥8.6% during the follow-up period, the risk of 3-point MACE (3p MACE, usually referring to cardiovascular death, non fatal myocardial infarction, and non fatal stroke) is significantly increased (HR = 1.79, 95% CI 1.16–2.79, *p* = 0.009) [65]. The hemoglobin glycation index (HGI), as a measure of the deviation between HbA1c and fasting blood glucose, has a higher predictive value for MACE (HR = 1.473, 95% CI 1.365–1.589, *p* < 0.001) [66].

Dyslipidemia and myocardial infarction: diabetes with dyslipidemia (characterized by high triglycerides and low high-density lipoprotein cholesterol (HDL-C)) significantly increases the risk of coronary heart disease, in which the elevated level of low-density lipoprotein cholesterol (LDL-C) is an independent risk factor for severe coronary heart disease (OR = 1.151, *p* = 0.046) [65]. Lipid metabolism disorder has existed in pre diabetes and is closely related to the accumulation of ectopic fat [64].

### 3.2 Heterogeneity of Fat Distribution and Cardiovascular Risk

The pathological impact of visceral fat is not limited to the release of inflammatory factors. Clinical imaging data show that obese T2DM patients have significantly increased EAT, and the increase in EAT is associated with a significant increase in coronary artery calcification score (CAC) [67, 68]. Its mechanism is closely related to the infiltration of proinflammatory factors (such as IL-6 and C-reactive protein CRP) secreted by EAT into the coronary artery, aggravating local inflammation and plaque instability [68].

These findings not only elucidate the strength of the association between GLMD and cardiovascular events but also provide critical insights for prevention and therapeutic strategies.

## 4. Precision Intervention Strategy: From Molecular Mechanism to Clinical Translation

The latest progress in precision medicine for diabetes provides an important reference for precise intervention of GLMD-related cardiovascular damage. By integrating individual biological, environmental and lifestyle information, multi-omics technologies (such as genomics, transcriptomics, proteomics, and metabolomics) can be used to more accurately identify patient subtypes and provide precise targets for the prevention and treatment of cardiovascular complications.

### 4.1 Core Drugs Targeting Metabolic Disorders: Beyond Glucose Lowering and Lipid Regulation

#### 4.1.1 SGLT2 Inhibitors (SGLT2i)

The mechanism of SGLT2i is to inhibit sodium-glucose cotransporter 2 (SGLT2) in the proximal tubule of the kidney and to promote urinary glucose excretion [69]. SGLT2i confers cardiovascular protection that is partly independent of glucose lowering. Their pleiotropic effects include: (1) enhancing myocardial energy efficiency by shifting substrate utilization toward ketone bodies and alleviating lipotoxicity [70]; (2) improving sodium balance and hemodynamics through natriuresis and diuresis, thereby reducing cardiac preload and afterload [69]; and (3) decreasing ventricular stiffness by attenuating coronary inflammation- achieved via reductions in EAT volume and suppression of its pro-inflammatory secretome [71]. These agents are indicated for patients with type 2 diabetes mellitus who have established ASCVD or are at high risk for ASCVD, for those with HF with reduced ejection fraction (HFrEF) or preserved ejection fraction (HFpEF), and for individuals with chronic kidney disease (CKD). Applicable populations: T2DM patients with ASCVD or high risk of ASCVD, HF (with reduced ejection fractionHFrEF/with preserved ejection fractionHFpEF ), and CKD [69]. Those with elevated N-terminal pro-B-type natriuretic peptide (NT-proBNP) may especially benefit from these agents [72, 73].

#### 4.1.2 GLP-1 Receptor Agonist (GLP-1RA)

GLP-1RAs work by activating GLP-1 receptors, thereby enhancing glucose-dependent insulin secretion, suppressing glucagon release, slowing gastric emptying, and increasing satiety. Their cardiovascular benefits are chiefly manifested by markedly lowering the risk of ASCVD events such as myocardial infarction and stroke-through anti-atherogenic actions that attenuate vascular inflammation, improve endothelial function, and stabilize plaques by shrinking the lipid-rich necrotic core and thickening the fibrous cap. The mechanism of GLP-1RA is to activate GLP-1 receptors to enhance glucose-dependent insulin secretion, inhibit glucagon, delay gastric emptying, and increase satiety. Cardiovascular benefits are mainly reflected in a significant decrease in the risk of ASCVD events (myocardial infarction, stroke): Anti-atherosclerosis benefits: Reduces vascular inflammation, improves endothelial function,and stabilizes plaques (reduces lipid necrotic core in plaques and increases fibrous cap thickness) [74].

#### 4.1.3 GRK-Biased Agonists

GRK-biased adrenergic agonists are a new class of drugs that bias G protein coupling. The GRK2 pathway of the receptor (GPCR) reduces cAMP production, thereby reducing cardiac side effects and effectively promoting muscle glucose uptake. This drug has shown good efficacy and safety in the treatment of type 2 diabetes and obesity, and is expected to become a new treatment option for cardiovascular complications of diabetes [75].

#### 4.1.4 TGR5 Agonists Targeting Lipotoxicity (Inhibiting CD36 Membrane Translocation) and ACBP Intervention (Improving Fatty Acid Metabolism/Myofilament Structure) Synergistically Alleviate Myocardial Lipid Overload

*TGR5* agonists: Activation of myocardial *TGR5* signaling can inhibit DHHC4-mediated *CD36* palmitoylation and membrane translocation, reduce myocardial fatty acid uptake, improve lipotoxicity and cardiac function, and provide a theoretical basis for the development of selective *TGR5* agonists to treat DbCM [34].

Targeted ACBP (acyl coenzyme A binding protein) intervention: the expression of ACBP in diabetic myocardium is up-regulated. ACBP not only exacerbates the disorder of fatty acid metabolism, but also disrupts muscle filament structure and reduces contractility by abnormally binding to the sarcomere protein Myosin Binding Protein C (MyBPC3). Myocardial specific knockout of ACBP can improve cardiac function in diabetic mice, suggesting that ACBP is a potential double effect target [46].

#### 4.1.5 Precision Blocking Strategy Targeting cGAS-STING Pathway

The cGAS-STING pathway is a “common inflammatory hub” for multi-organ complications of diabetes (such as cardiomyopathy, nephropathy, and retinopathy), and its inhibitors show potential for precision intervention [45].

C-176 (*STING* dimerization inhibitor): significantly reduced myocardial IFN-β and collagen deposition in db/db diabetic mouse model [45].

H-151 (*STING* covalent inhibitor): In the myocardial infarction (MI) model (non-diabetic cardiomyopathy model), by specifically inhibiting the *cGAS*-*STING*-IRF3 pathway in infiltrating macrophages, it significantly reduced the type I interferon response, thereby: improving myocardial contractile function, and reducing cardiac fibrosis [76].

### 4.2 Precise Screening and Risk Stratification: Biomarkers and AI Empowerment

#### 4.2.1 NT-proBNP-Guided Cardiovascular Risk Management

NT-proBNP is a marker of myocardial wall stress and is closely related to the occurrence and prognosis of HF. Incorporating NT-proBNP into cardiovascular risk assessment models (such as the Systemic Coronary Risk Assessment 2 (SCORE2) model) can significantly improve risk prediction capabilities, especially in identifying high-risk populations [77].

#### 4.2.2 Metabolic Memory-Related Markers

Plasma levels of the miR-15-16 cluster in small extracellular vesicles (sEVs), and are closely associated with diabetes-related metabolic memory and the risk of cardiac damage. These plasma sEV miR-15-16 cluster levels demonstrate a significant correlation with cardiac dysfunction in diabetic patients-independent of blood glucose or HbA1c levels-and represent a promising novel biomarker for assessing the risk of metabolic memory-associated cardiac injury [51].

#### 4.2.3 Integration of Multimodal Data and Artificial Intelligence (AI)

Machine learning enables identification of high-risk individuals for cardiovascular events among diabetic patients through analysis of extensive clinical datasets. By integrating clinical, laboratory, and imaging data within ML algorithms, accurate predictive models can be developed to enhance cardiovascular risk stratification [78]. Deep neural networks (DNNs) have shown significant promise in this domain: one study successfully predicted 1-year all-cause mortality using only 12-lead electrocardiogram (ECG) voltage data via a DNN architecture, demonstrating robust performance across large-scale validation [79] . This DNN-based approach not only improves prediction accuracy but also offers a powerful tool for early cardiovascular disease screening and intervention.

### 4.3 Intervention Targeting Gut Flora and Metabolic Memory

Microbial regulation: Dietary intervention (such as the Mediterranean diet): The Mediterranean diet, which is rich in fiber, polyphenols, and unsaturated fatty acids, can promote the growth of beneficial bacteria (SCFA-producing bacteria), increase SCFA levels, and reduce serum inflammatory markers (such as high-sensitivity C-reactive protein hs-CRP) [80, 81]. Its cardiovascular protective effect is partly achieved by improving the microbiota.

Probiotics/prebiotics/synbiotics: Specific strains (such as certain bifidobacteria, lactobacilli) or prebiotics (such as inulin, oligofructose) have shown the potential to improve lipid metabolism, reduce TMAO, and reduce inflammation in animal and some human studies [82, 83].

Selective inhibition of harmful pathways: The development of drugs that inhibit bacterial TMA cleavage enzymes or host FMO3 activity to reduce TMAO levels is an active research direction [84]. Breakthroughs have been made in the clinical transformation of targeted TMAO, and early clinical studies of FMO3 inhibitors have shown potential [85], but long-term efficacy requires phase III trials to verify.

Metabolic memory intervention: Early intensive blood sugar control is the key time window for blocking the formation of the O-GlcNAc/CaMKIIδ/STAT1/*miR-15-16* loop. The development of O-GlcNAc transferase (OGT) inhibitors or CaMKIIδ inhibitors is a potential “metabolic memory erase” strategy, which is currently in the preclinical exploration stage [51].

Application of multi-omics technology in precision intervention: Multi-omics technology (including genomics, transcriptomics, proteomics and metabolomics) provides new tools for precision intervention of cardiovascular diseases. By integrating multi-omics data, the molecular network of disease progression can be revealed, new biomarkers and therapeutic targets can be identified, and personalized treatment strategies can be achieved [86].

## 5. Synthesis of Advances and Unresolved Challenges

To consolidate recent breakthroughs and translational needs, Table 1 (Ref. [26, 27, 42, 45, 46, 51, 56, 57, 58, 59, 71, 75, 77]) summarizes key advances and critical research gaps for the four primary cardiovascular complications of GLMD.

**Table 1.** **Advances and research gaps in GLMD-related cardiovascular complications (2018–2025)**.

Disease	Key advances	Identified research gaps
ASCVD	Role of Lp(a) in thrombosis (Wong *et al*. 2024) [26]	Lipoprotein dysfunction (Lp(a) thrombosis + HDL impairment) drives plaque instability
	HDL dysfunction promotes plaque instability (Madaudo *et al*. 2024) [27]	Long-term effects of EAT modulation on plaque stability
	SGLT2i reduces EAT inflammation (Bao *et al*. 2025) [71]	
DbCM	Mitochondrial autophagy impairment amplifies lipotoxicity (Dewanjee *et al*. 2021) [42]	Tissue-specific modulation of mitochondrial quality control
	cGAS-STING pathway links lipotoxicity to inflammation (He *et al*. 2024) [45]	Safe delivery of STING inhibitors to cardiomyocytes
	ACBP disrupts sarcomere structure and exacerbates lipotoxicity (Wu *et al*. 2025) [46]	Clinical validation of ACBP inhibitors
HF	sEV miR-15-16 as metabolic memory biomarker (Ding *et al*. 2025) [51]	Dynamic monitoring of sEV biomarkers
	GRK-biased agonists improve myocardial glucose uptake (Motso *et al*. 2025) [75]	Pharmacokinetic optimization of GRK agonists for cardiac specificity
	NT-proBNP enhances cardiovascular risk prediction (Lehmacher *et al*. 2022) [77]	AI-integrated risk prediction for HF phenotypes
CAN	FFA and cytokines activate neuronal TLR4-NF-κB signaling (Meng *et al*. 2022) [56]	Early biomarkers for subclinical CAN
	Autoimmune diabetes increases CAN susceptibility (Risi *et al*. 2025) [57]	Neuron-targeted delivery of anti-inflammatory agents
	Gut microbiota metabolites (e.g., TMAO) drive endothelial injury and autonomic imbalance (Wen *et al*. 2022 [58], Tanase *et al*. 2020 [59])	Clinical trials of microbiota-directed interventions

DbCM, diabetic cardiomyopathy; HDL, high-density lipoprotein; ACBP, acyl-coenzyme A binding protein.

## 6. Future Research Directions

### 6.1 Molecular Subtyping of GLMD Heterogeneity

Define high-risk GLMD subtypes through integrated multi-omics (genomics, metabolomics, microbiome) and deep phenotyping (imaging, dynamic glucose monitoring). Key subtypes include: nflammation-dominant (elevated CRP/IL-6, EAT thickening); Severe lipotoxicity (myocardial lipid deposition, ACBP/*CD36* overexpression); Metabolic memory-sensitive (persistently high sEV *miR-15-16* levels); Dysbiosis-driven (reduced SCFA-producing bacteria, elevated TMAO-generating microbiota).

### 6.2 Tissue-Targeted Delivery Systems

Many promising therapeutic targets (such as CD36 and STING within cardiomyocytes, and CaMKIIa within endothelial cells) are located in specific organelles or cell types. Systemic drug administration often faces challenges including off-target effects, dose-limiting toxicity, or difficulties in achieving effective concentrations at the target site. Developing heart/vascular-specific targeted nanocarriers (e.g., liposomes, engineered exosome carriers) or adeno- associated virus (AAV)-based gene therapy vectors is crucial for enhancing their efficacy and reducing side effects. For example, overcoming the myocardial-specific delivery bottleneck is essential for cGAS-STING targeted therapy: Existing inhibitors (e.g., C-176) administered systemically readily interfere with immune surveillance functions, potentially increasing the risk of infection. Solutions include developing myocardial-targeted liposomal encapsulation technology that utilizes highly expressed proteins in cardiomyocytes (such as cardiac troponin I (cTnI) antibody modification) to achieve drug enrichment.

### 6.3 Dynamic Multi-Omics-AI Risk Prediction Models

Moving beyond single or a few biomarkers, we integrate static (genetic, epigenetic) and dynamic (transcriptomic, proteomic, metabolomic, microbiome, radiomic, continuous glucose/physiological monitoring) data streams. Machine learning and deep learning are leveraged to construct dynamically updatable, individualized cardiovascular risk prediction models. For instance: Integrating ECG-AI features (derived from electrocardiogram-artificial intelligence analysis), metabolic memory markers (e.g., sEV miR-15-16 clusters), radiomics (EAT volume), and continuous glucose monitoring (CGM) data enables the development of significantly more accurate dynamic risk stratification models.

## 7. Conclusion

GLMD drives multi-organ cardiovascular injury—encompassing ASCVD, DbCM, HF, and CAN—through interconnected molecular cascades: Vascular inflammation via AGEs-RAGE/NF-κB axis and lipoprotein dysfunction (e.g., Lp(a)-mediated thrombosis, HDL impairment). Myocardial lipotoxicity fueled by *CD36*-mediated fatty acid overload, mitochondrial ROS/mtDNA leakage, and *cGAS*-*STING*-driven inflammation. Metabolic memory sustained by O-GlcNAc/CaMKIIδ/STAT1/*miR-15-16* loops in endothelial sEVs. Gut-heart axis disruption via TMAO (thrombosis/endothelial injury) and diminished SCFAs. Neuro-cardiovascular dysregulation from visceral fat inflammation and autoimmune-triggered CAN. Emerging mechanisms—particularly *cGAS*-*STING* as a unifying inflammatory hub and ACBP/*TGR5*-modulated lipotoxicity—reveal novel targets for precision interventions: NT-proBNP-guided SGLT2i/GLP-1RA therapy optimizes cardiorenal protection in high-risk subsets. *TGR5* agonists and ACBP inhibition synergistically reverse myocardial lipid overload. O-GlcNAc/CaMKIIδ pathway blockade disrupts metabolic memory. Microbiota-directed strategies (FMO3 inhibitors, TMA lyase antagonists) reduce TMAO and improve endothelial function in early trials. Challenges persist: Heterogeneity: Molecular subtyping (e.g., inflammation-dominant vs. lipotoxicity-severe GLMD) is needed. Delivery limitations: Tissue-targeted systems (e.g., cardiac-tropic exosomes for *miR-15-16* inhibitors) require development. Dynamic prediction: AI-integrated multi-omics models (Section 4.3) must be validated prospectively. Future translation hinges on: Adipose/vascular-targeted therapies (e.g., HIF-1α inhibitors). Gene-editing approaches (e.g., CD36 modulation via AAV). Real-world evidence for microbiota precision interventions.
